# Bone marrow mesenchymal stem cells alleviate neurological dysfunction by reducing autophagy damage via downregulation of SYNPO2 in neonatal hypoxic–ischemic encephalopathy rats

**DOI:** 10.1038/s41419-025-07439-w

**Published:** 2025-02-25

**Authors:** Lu- Lu Xue, Jie Cheng, Ruo-Lan Du, Bo-Yan Luo, Li Chen, Qiu-Xia Xiao, Hong-Su Zhou, Hong-Qing She, Shi-Feng Wang, Ting-Bao Chen, Chang-Yan Hu, Yu-Qi He, Ting-Hua Wang, Liu-Lin Xiong

**Affiliations:** 1 Department of Anesthesiology, The Third Affiliated Hospital of Zunyi Medical University (The First People’s Hospital of Zunyi), Zunyi, Guizhou China; 2 Center for Reproductive Medicine, Renji Hospital, School of Medicine, Shanghai Jiao Tong University, Shanghai, China; 3 School of Pharmacy, Zunyi Medical University, Zunyi, Guizhou China; 4 Department of Neurosurgery, Institute of Neurological Disease, West China Hospital, Sichuan University, Chengdu, Sichuan China; 5 Institute of Neuroscience, Kunming Medical University, Kunming, Yunnan China

**Keywords:** Mesenchymal stem cells, Encephalopathy

## Abstract

Neonatal hypoxic-ischemic encephalopathy (HIE) is worsened by autophagy-induced neuronal damage, with SYNPO2 playing a key role in this process. This study investigates the involvement of SYNPO2 in neuronal autophagy and explores the potential of bone marrow mesenchymal stem cells (BMSCs) to alleviate HIE-induced dysfunction by inhibiting SYNPO2-mediated autophagy. Using in vitro and in vivo neonatal HIE models, we observed an upregulation of SYNPO2 expression, accompanied by increased neuronal injury and aggregation of autophagy-related proteins. Intervention with BMSCs effectively reduced SYNPO2 expression, and SYNPO2 depression mitigated neuroautophagic damage and improved neurological dysfunctions. Moreover, SYNPO2 overexpression exacerbated neuroautophagy despite BMSC treatment, while SYNPO2 depletion notably reduced neuroautophagic damage and alleviated cognitive impairments, retaining the neuroprotective efficacy of BMSC treatment. These findings confirm the role of BMSCs in attenuating HIE injury by suppressing neuroautophagy and provide insights into the mechanistic involvement of SYNPO2. Ultimately, this study identifies SYNPO2 as a novel therapeutic target for neonatal HIE and supports the clinical potential of BMSCs in HIE management.

## Introduction

Hypoxic-ischemic encephalopathy (HIE) is one of the most common and severe neonatal neurological diseases, caused by hypoxia, cerebral blood flow reduction, or transient interruptions in cerebral perfusion [1]. This type of brain damage is closely associated with chronic neurological impairments, including motor dysfunction and memory deficits, affecting millions of newborns annually [2, 3]. Current clinical treatments for HIE, such as cardiopulmonary bypass and neuroprotective drugs, face limitations in terms of timing and efficacy [4, 5]. Consequently, there remains a pressing need to explore highly selective and precise therapeutic approaches to enhance treatment outcomes and mitigate neurological damage in children with HIE. The mechanisms underlying HIE involve multiple pathways, including oxidative stress [6], apoptosis, and autophagy [7, 8]. Among these, autophagy is a particularly critical pathological process. While autophagy serves as a cellular housekeeping mechanism, excessive autophagy can trigger neuronal injury and apoptosis, exacerbating inflammation and brain damage [9, 10]. Studies have shown that neonatal HIE disrupts autophagy flux, intensifying brain injury and leading to cognitive and memory deficits during adolescence [11, 12]. As such, autophagy inhibition has emerged as a potential neuroprotective strategy for addressing neonatal HIE-induced brain damage [13, 14]. Moreover, pharmacological or gene therapy-mediated regulation of autophagy has been found to alleviate HIE-induced neuronal injury and dysfunction [15, 16]. This suggests that targeting genes and proteins involved in autophagy may offer novel therapeutic opportunities for HIE treatment.

Recent studies highlight the significant potential of stem cell-based therapies in ameliorating neurological deficits following HIE [17, 18]. Various types of multipotent stem cells, including neural stem cells (NSCs) [19, 20] and mesenchymal stem cells (MSCs) [21, 22], have shown promising results in preclinical models. Bone marrow mesenchymal stem cells (BMSCs) are particularly noteworthy due to their low immunogenicity and lack of ethical concerns [23]. BMSCs have garnered increasing attention in regenerative medicine and neurology [21, 24], for their ability to enhance the neuronal survival, reduce neuroinflammation caused by HIE, and repair motor cortical damage [25–27]. Additionally, BMSCs have been reported to effectively alleviate HIE-related brain damage by regulating autophagy, suggesting BMSC-based therapies hold significant therapeutic potential for treating HIE by regulating autophagy [25, 28, 29]. However, the precise mechanisms by which BMSCs regulate autophagy remain unclear.

Synaptopodin-2 (SYNPO2) is a functional protein predominantly expressed in the heart, smooth muscle, skeletal muscle, and neurons. SYNPO2 plays a pivotal role in regulating autophagy, particularly in muscle cells [30, 31], and its homolog synaptopodin (SYNPO) expressed in the synaptic structure of neurons, was primarily correlated with neuronal morphology and function [32–34]. SYNPO2 maintains muscle function under stress via chaperone-assisted selective autophagy (CASA) [35]. SYNPO2’s regulatory function of autophagy depends on its PDZ domain, which enables the recruitment of sequestosome-1 (SQSTM1) and interaction with Bcl-2-associated athanogene-3 (BAG3) to promote autophagy [36, 37]. In neurons, SYNPO supports autophagic flux in the postsynaptic compartments, contributing to the clearance of phosphorylated microtubule-related protein Tau in dendritic spines [38]. Although the role of SYNPO2 in HIE-induced neuroautophagy and neuronal injury remains to be elucidated, it represents a potential new mechanism and therapeutic target for HIE.

In this study, we employed SYNPO2 lentiviral infection and BMSC-based treatments on cortical neurons and animal models of HIE. Through morphological, molecular, and behavioral experiments, we aimed to elucidate the role of SYNPO2 in HIE-induced neuroautophagy and neurological impairments, providing a new therapeutic target and rationale for the clinical application of BMSCs in HIE management.

## Materials and methods

### Animal care and grouping

Neonatal Sprague Dawley (SD) rats (both male and female, weighing 10–19 g) were obtained from the Experimental Animal Center of Zunyi Medical University. Neonatal SD rats were used for different experimental purposes as follows: 1-day-old rats were used for cortical primary neuron culture, 3-day-old rats for lentiviral injections, 7-day-old rats for HIE modeling, and 30-day-old rats for BMSC extraction. Animals were excluded from the study if they exhibited persistent weight loss after modeling, failed ligation of the right common carotid artery during the modeling process, or significant individual variability that resulted in an inability to replicate the expected pathological features of the HIE model. All animal experiments were approved by the Experimental Animal Ethics Committee of Zunyi Medical University ([2020]2-097), and conducted in compliance with *Guide for the Care and Use of Laboratory Animals* published by the National Institutes of Health. Animals were housed in cages with ad libitum access to food and water, under controlled conditions of 26 ± 0.5 °C and 65 ± 5% humidity. To ensure unbiased results, measurements and observations were repeated three times and performed by researchers blinded to the group assignments. Sample sizes were estimated by MedSci Sample Size tools with comparison of the mean of two independent samples: α = 0.05, power=0.8. Animals were randomly assigned into the following groups using simple random sampling: Sham, HIE (HIE + PBS, HIE+BMSCs, HIE+shNC, HIE+shSYNPO2, HIE+BMSCs+OE-NC, HIE+BMSCs+OE, and HIE+BMSCs+dPDZ groups. Brain tissues were collected at 24 hours after the hypoxic-ischemic insult for immunostaining experiments, at 39 days after injury for Nissl and HE staining. To minimize bias, all experiments in this study were conducted in a double-blind manner, wherein both the researchers performing the procedures and those analyzing the data were unaware of the group assignments or treatment conditions.

### Primary cortical neuron culture

Cortical tissues were obtained from 1-day-old newborn SD rats, which were dissected, minced, and digested with 0.25% trypsin for 10–15 minutes (min) at 37 °C. After digestion, the samples were eluted using a basal medium containing 10% fetal bovine serum (FBS), filtered 3 times with a 70 µm cell sieve, and centrifuged at 1500 rpm for 5 min at room temperature. Afterward, the supernatant was discarded, and the basal culture medium was added to prepare a single-cell suspension. Immediately, cells at a density of approximately 5 × 10^5^/ml were seeded on the poly-L-lysine and laminin-coated coverslips and cultured at 37 °C in 5% CO_2_ and 95% air. After 4 hours, the basal culture medium was replaced with a neuron culture medium. The basal culture medium consisted of high-glucose DMEM (Biosharp, Canada), 10% FBS (Gibco, USA), 1% penicillin-streptomicin (PS) (Cyagen, USA, California). Neuron culture medium was composed of Neurobasal-A medium (Gibco, USA), 2% B27 (Gibco, USA), 1% L-glutamin (Cyagen, USA), and 0.5% PS (Cyagen, USA).

### Lentiviral infection in vitro and in vivo

On day 3, the neuron culture medium was replaced with a labeling medium consisting of the neuron culture medium (without PS), transfection reagent A, and lentivirus. After 8 hours, the labeling medium was completely replaced with the neuron culture medium (without glutamine). Fluorescence imaging was performed 4 days post-infection. Lentiviruses were procured from Shanghai Genechem Co., Ltd. The multiplicity of infection (MOI) is 5. This study included the following lentivirus: SYNPO2 interference controls (shNC, 5 × 10^8^ TU/mL), SYNPO2 interference (shSYNPO2, 3 × 10^9^ TU/mL), SYNPO2 overexpression controls (OE-NC, 2.5 × 10^8^ TU/mL), SYNPO2 overexpression (OE, 2.5 × 10^9^ TU/mL), SYNPO2-dPDZ deletion mutant (dPDZ, 1.5 × 10^9^ TU/mL).

For lentiviral injection in vivo, rats on postnatal day 3 were anesthetized with isoflurane for lentiviral transplantation. A total of 3 μL of lentivirus (7.5 × 10^5^ TU) was injected into the lateral ventricle at a speed of 1 μL/min using the following stereotaxic coordinates: anterior-posterior (AP): −0.5 mm, medial-lateral (ML): −1.2 mm, and dorsal-ventral (DV): −2.0 mm. After completing the injection, the micro-syringe was left in place for 3 min before being carefully withdrawn. The scalp was then sutured, and the area was disinfected with iodophor. Following the procedure, the neonatal rats were returned to the cages once they had fully regained consciousness.

### Establishment of oxygen-glucose deprivation (OGD) model

On postnatal day 7, primary cortical neurons were subjected to OGD to mimic hypoxic-ischemic conditions in vitro. According to previous studies [14], the neuron culture medium (without glutamine) was completely substituted by the glucose-free DMEM medium. Subsequently, cortical neurons were placed into a hypoxia chamber with 5% CO_2_, 95% N_2_, and 1% O_2_ at 37 °C for 40 min culture. Post-hypoxia, the glucose-free DMEM medium was replaced with the neuron culture medium and then cultured in a 37 °C, 5% CO_2_ cell incubator for 24 hours.

### BMSC-conditioned medium (CM) intervention

BMSCs were isolated from 30-day-old SD rats following cervical dislocation and disinfection in 75% ethanol. The femurs and tibias were carefully dissected, cleaned, and flushed with a complete BMSC culture medium to extract bone marrow. The collected bone marrow suspension was cultured at 37 °C in 5% CO_2_, with the medium changed 48 hours after initial plating and subsequently every two days to maintain optimal growth conditions. CM was prepared from the culture medium of the 2^nd^–5^th^ generation BMSCs. The medium was filtered through a 220 nm filter, and then concentrated with a 3 kDa ultrafiltration tube in the centrifuge at 3000 *g* for 1 hour. Immediately after OGD, 2 × CM was added to the primary cortical neurons of the corresponding group for a 24-hour intervention.

### Immunofluorescence staining in vitro

Cortical neurons were harvested 24 hours post-OGD and washed 3 times with PBS, followed by fixation in 4% paraformaldehyde for 15 min. Afterwards, these cells were washed 3 times with PBS, 5 min/time. Neurons were permeabilized and blocked with 0.3% TritonX-100 and 5% goat serum at 37 °C for 30 min. Then, primary antibodies were added to incubate at 4 °C overnight (16–18 hours): P62 (rabbit, 1:200, 8420-1 AP, Proteintech), LC3B (rabbit, 1:200, 192890, Abcam), Cleaved-caspase3 (C-cas3, rabbit, 1:100, 9664 s, CST), and TUJ1 (mouse, 1:400, ab78078, Abcam). Subsequently, the samples were washed and incubated with secondary antibodies (Goat anti-rabbit IgG 647, 1:400, ab150079, Abcam; Goat anti-mouse IgG 488, 1:400, A23220, Abbkine) at room temperature for 3 hours. For TUNEL staining, samples were incubated at 37 °C for 1 hour. The secondary antibodies for TUNEL staining were prepared by mixing TdT enzyme and fluorescent labeling solution 647 in a 1:9 ratio. After PBS washes, DAPI (Beyotime, 1:3000) was applied for nuclear staining, and the anti-fluorescence quenching agent was used for sealing. Imaging was performed with a Leica confocal microscope, and ImageJ software (v.1.8.0) was used for measuring axon length and indicated positive neurons. Apoptosis rate (%) was calculated by counting the number of Tuj1^+^TUNEL^+^ or C-cas3^+^/Tuj1^+^ cells and normalizing them to the number of Tuj1^+^ cells (which marks total neuron count) in the same field of view. Autophagic neurons (%) were measured by counting the number of LC3B^+^/Tuj1^+^ or P62^+^/Tuj1^+^ cells and normalizing them to the number of Tuj1^+^ cells (which marks total neuron count) in the same field of view.

### Cell viability assay (CCK8)

CCK8 was used to measure cell viability 24 hours after OGD based on the manufacturer’s instructions. The 96-well plate was prepared with 3 wells designated as the blank group, containing 90 μL of neuron culture medium and 10 μL of CCK8 reagent (Bioss, China). For the experiment group, 90 μL of neuron culture medium and 10 μL of CCK8 solution were added to the corresponding wells. The plate was incubated at 37 °C for 2 hours, and then the optical density (OD) was measured at 450 nm. Cell viability was calculated as: cell viability=OD value of the experimental group-OD value of control group.

### Western blot (WB)

WB was used to detect the levels of target proteins in OGD neurons on culturing 8 days. Briefly, the total protein was extracted from the cultured cells and dissolved in 15% SDS-polyacrylamide gel. Then, the protein was separated into protein bands by electrophoresis buffer at 80 volts for 20 min and at 120 volts for 30 min. Afterward, the transfer buffer was added to transfer the protein on the SDS-PAGE gel to the PVDF membrane at a rate of 350 mA for 4 hours. The PVDF membrane was blocked with 5% skim milk at room temperature for 1–2 hours and then incubated with the primary antibodies SYNPO2 (rabbit, 1: 1000, 25453-1 AP, Proteintech), P62 (rabbit, 1: 1000, 8420-1 AP, Proteintech), Beclin1 (rabbit, 1: 1000, 207612, Abcam), AIFM1 (rabbit, 1: 1000, bs0037R, Bioss), ATG12 (rabbit, 1: 1000, 155589, Abcam), LC3B (rabbit, 1: 1000, 192890, Abcam), GAPDH (rabbit, 1: 1000, 6004, Proteintech) and β-actin (mouse, 1:5000, T0022, Affinity) in 3% BSA at 4 °C overnight. After incubation, the membrane was washed with TBST (TBS containing 0.2% Tween-20) for 4 times, incubated with Horseradish peroxidase (HRP) (Goat anti-rabbit, 1:5000, A21020, Abbkine; Goat anti-mouse, 1:5000, A21010, Abbkine) for 1 hour at room temperature and then wash with TBST for 4 times. Finally, the A and B luminescent solutions of the ECL chemiluminescence substrate kit were diluted and mixed in proportion (1:1) for development, and images were collected in the gel imaging instrument. ImageJ (v.1.8.0) was used to measure the gray value.

### HIE model in newborn SD rats

Hypoxia-ischemia procedure was conducted as described previously [39]. Briefly, 4 days after being injected with lentivirus at postnatal day 3, rats on postnatal day 7 were anesthetized with isoflurane, and the skin was cut longitudinally about 1 cm in the center of the neck to expose the right common carotid artery (CCA). Then blood vessels were ligated by electrocoagulation, and the subcutaneous tissue and skin were stitched. Last, the rats were taken back to their mother to be restored for 1 hour. Afterwards, the postoperative neonatal rats were put in the anoxic box for anoxia (8% O_2_ + 92% N_2_, temperature: 36.6 °C, humidity: 60–70%, time: 1.5 hours). Rats in the Sham operation group underwent the same anesthesia and arterial exposure without arterial occlusion and hypoxia.

### BMSC transplantation

Thirty min after HIE modeling, rats were transplanted with 2 × 10^5^/5 μL BMSCs in the lateral ventricle at an injection rate of 1 μL/min. The control group was transplanted with an equal amount of PBS treatment. The injection was completed and stayed for 3 min, the micro-syringe was slowly taken out. Then, the subcutaneous tissue and skin were sutured and disinfected with iodophor, and put into the cages after waking up. The SD rats transplanted with BMSCs were intraperitoneally injected with cyclosporine A (10 mg/kg) every day until sampling.

### Animal sampling harvest

After the rats were anesthetized with isoflurane, their chest cavity was opened to expose the heart. A perfusion tube was inserted from the apex to the ascending aorta. The 0.9% sodium chloride was injected for perfusion. Afterwards, the rats were perfused with 4% paraformaldehyde fixative to the body stiffness. The brain tissues were taken out and fixed in 4% paraformaldehyde. After 24–48 hours, the sample was washed with PBS for 1–2 times. The brain tissues were respectively dehydrated in 10%, 20%, and 30% sucrose solution. It was taken out, dried, and put into liquid nitrogen for quick freezing. Subsequently, the samples were embedded in OCT and stored at −80 °C for frozen sectioning. The slices were labeled with the date of the tissue name and then baked for 2 hours in a 37 °C warmer. Finally, they were stored in a -20 °C refrigerator. For molecular biology detection experiments, brain tissues were harvested after perfusion with 0.9% sodium chloride solution, and stored at -80 °C.

### Quantitative real-time PCR (RT-qPCR)

The brain tissues were harvested at 24 hours after HIE modeling and neurons were collected 24 hours after OGD modeling. Total RNA was extracted using triazole reagent (Takara, Japan), and then was reverse transcribed into cDNA using a PCR kit (Takara). RT-qPCR analysis was performed using a real-time fluorescent quantitative PCR system (Bio-Rad, USA, California) according to the manufacturer’s instructions. GAPDH was used as an internal reference. The 2^−ΔΔCt^ method is used for data processing. Primer sequences are listed in Table 1.

**Table 1:** Primer sequences.

Gene	Species	Forward (5’-3’)	Reverse (5’-3’)
GAPDH	Rat	GACATGCCGCCTGGAGAAAC	AGCCCAGGATGCCCTTTAGT
SYNPO2	Rat	TGACAAGTCCCATCCCTGAC	TCTCATCGCGGGAAGCTATT

### Multiple immunohistochemical staining

Brain tissues were collected at 24 hours after the hypoxic-ischemic insult, and sections were slowly rewarmed at room temperature and then baked at 37 °C for 30 min and repaired with 1 × sodium citrate high-temperature antigen for 3 min. After antigen-repairing with sodium citrate, sections were incubated with H_2_O_2_ for 10 min and blockaded with 5% goat serum and 0.3% Triton X-100. Subsequently, the sections were supplemented with primary antibodies against LAMP1 (rabbit, 1:200, ab24170, Abcam), SQSTM1 (rabbit, 1:200, a19700, Abclonal), LC3B (rabbit, 1:200, ab192890, Abcam) for 18 hours at 4 °C. Rinsed in PBST for 8 times, secondary antibodies (Goat anti-rabbit, MaxVision-HRP, ab150079, Abcam) were added for incubation at room temperature for 10 min, followed by washes with PBST and 10 min-incubation with TSAPlus fluorescent enhancement dye (iF488-Tyramide, Servicebio, 1:1000). Whereafter, these sections underwent antigen repairing again with sodium citrate and incubation with H_2_O_2_ for 10 min, which were then blocked with 5% goat serum and 0.3% Triton X-100. Primary antibodies against NEUN (mouse, 1:800, ab104224, Abcam) were added respectively to be incubated for 18 hours at 4 °C. After that, incubation with secondary antibodies (Goat anti-mouse, MaxVision-HRP, A23220, Abbkine) were continued at room temperature for 10 min, followed by PBST washes and 10 min-incubation with TSAPlus fluorescent enhancer (iF594-Tyramide, Servicebio, 1:1000). After counterstaining with DAPI for 10 min, sections were sealed with anti-fluorescent quenching agent. Images were captured under the two-photon confocal microscope (Leica TCS SP8 DIVE).

### Transmission electron microscope (TEM)

Twenty-four hours after the HIE model, the rats were anesthetized with isoflurane and the brain tissue was harvested. Pre-cooled 500 μL of 3.5% glutaraldehyde was dripped to the brain tissue for superficial fixation. A 1 × 1 × 1 cm cerebral cortex was extracted and stored in 1.5 mL of pre-cooled 3.5% glutaraldehyde at 4° C for 7 days. The samples were dehydrated using a gradient ethanol series and embedded in 618 epoxy resin. Ultrathin sections were prepared and stained with 2% uranyl acetate and lead citrate. Autophagy in brain tissue was observed using JEM-1400 Flash TEM (JEOL, China).

### Forelimb grip test, righting test, geotaxis test, and climbing test

Behavioral tests were conducted to assess early muscle function and motor coordination in rats following HIE. Forelimb grip test: At 24 hours and 7 days post-HIE, the forelimb grip strength of the rats was evaluated to measure their muscle function [14]. Righting test: At 7 days post-HIE, the time required for the rats to right themselves when placed on their backs on a flat table was recorded [40]. Geotaxis test: In this test, rats were placed head down on a 45° incline, and the time taken to turn 180° upward was measured [41]. Climbing test: Rats were placed face down on a 45° slope, and the time required to turn 180° upward and climb a designated line was recorded [39]. These tests provided a comprehensive evaluation of the motor coordination and muscle functionality of the rats in the early stages of recovery after brain injury.

### Rotarod test

This rotarod test was conducted to assess the coordination and motor ability of rats after HIE damage [42]. Rats were initially trained on a rotating tester at a constant speed of 10 revolutions per minute (rpm) for 3 consecutive days, with 3 training sessions per day, each lasting 10 min. The formal experiment was performed the next day after the completion of the training. During test, rats were placed on the rotarod, which accelerated from 0 to 30 rpm in 180 seconds. Once the speed reached 30 rpm, it remained constant, and the time until each rat fell from the rod was recorded. Each rat underwent 3 consecutive trials, and the average time (in seconds) was calculated to assess their performance.

### Open field test

The open field test [43] was used to evaluate the effects of HIE injury on the autonomic behavior, exploratory behavior, and tension of rats. The experimental device consisted of an open field box and an automatic data acquisition and processing system (SuperMaze V2.0, Xinruan Info Tech Ltd., China). Prior to testing, rats were acclimated to an open field environment over 3 consecutive days (10 min/day). On the testing day, rats were removed from their cages with their backs toward the experimenter, and placed in the center of the open field. Video tracking software system automatically recorded the number of center entries and time spent grooming during a total of 10-min tests. To ensure the reliability of the results, the open field box needs to be cleaned with 70% ethanol after each trial to prevent any residual odors or substances from affecting subsequent tests. Each animal underwent testing once in the morning, midday, and evening.

### Y-maze test

Y-maze test [44] was used to determine the spatial recognition and memory ability after HIE. Y-maze consists of 3 arms, each arm forming a 120° angle, and measuring 30 cm × 8 cm × 15 cm (length × width × height). The experiment was divided into two periods. Acquisition period: After 24 hours of fasting, each rat was placed in the starting arm, with the error arm closed and food placed in the food arm. Rats in each group were allowed to freely explore in the starting arm and the food arm for 3 min per trial. Training was conducted 12 times for each rat, and during this phase, the number of correct and incorrect responses was not recorded. Formal experiment: all 3 arms of the Y-maze were opened, and the animals were free to move for 5 min. The number of entries into the food arm was recorded with a video camera and analyzed by SuperMaze V2.0 (Xinruan Info Tech Ltd.).

### Morris water maze test

Morris water maze was used to assess the effect of brain injury on spatial learning and memory after HIE [45]. The experiment spanned 6 days, including 5 days of hidden platform training and 1 day of spatial exploration experiment. The test was carried out in a circular pool divided into four equal quadrants, with black ink added to obscure the platform. A hidden black platform was positioned 1 cm below the water surface in the third quadrant. During the hidden platform training, rats were placed in the pool, and their latency to locate the platform was recorded. Each rat was trained continuously for 5 days, starting from each of the four quadrants in turn. If a rat failed to find the platform within 90 seconds, they would be guided to the platform and allowed to remain there for 5 seconds by laboratory staff. On the sixth day, the spatial exploration test was conducted by removing the platform. Rats were placed in the pool from the first quadrant, and the number of times they crossed the target area, the latency to find the platform, along with the trajectory traveled, were recorded. After each trial, rats were dried and put back into their cages to ensure comfort and safety.

### Hematoxylin and eosin (HE) staining

Brain tissues collected at 39 days after HIE modeling were washed with PBS and stained with hematoxylin and eosin (Solarbio, China). Afterward, it was dehydrated with gradient ethanol, soaked with xylene, and finally sealed with neutral resin. The cerebral neuronal damage was observed and photographed by an optical microscope (Ningbo Yu Shun, China).

### Nissl staining

Brain tissues were harvested at 39 days after HIE modeling for Nissl staining. The prepared brain sections were washed three times with PBS, and the Nissl staining solution (Beyotime) was added dropwise. Each brain tissue was 30–40 μL and allowed to stand at room temperature for 4 min. The sections were taken out and placed in distilled water for 1 min, then dehydrated by gradient concentrations of ethanol, soaked with xylene, and finally sealed with a neutral resin. Neuronal cell death in brain sections on 39 days after HIE was observed and photographed using an optical microscope (Ningbo Yu Shun, China).

### Statistical analysis

Data visualization was performed using GraphPad Prism 8.0 software, and all statistical analyses were conducted using the Statistical Package for the Social Sciences (SPSS 26.0). The Shapiro-Wilk test was applied to assess the normality of the data distribution. For normally distributed data, a two-sided unpaired independent sample *t*-test was used to compare two groups, while two-sided unpaired one-way analysis of variance (ANOVA) followed by Least Significant Difference (LSD) (equal variances) or Dunnett’s post (unequal variances) hoc tests were employed for comparisons among multiple groups. For non-normally distributed data, non-parametric statistical methods were applied. Specifically, the Mann-Whitney U test was used for comparisons between two groups, and the Kruskal-Wallis test was applied for comparisons among three or more groups. If the Kruskal-Wallis test indicated significant differences, post hoc pairwise comparisons were performed using Dunn’s method. All numerical variables are presented as mean ± standard deviation (Mean ± SD). A p-value < 0.05 was considered statistically significant.

## Results

### CM alleviated autophagic injury in cortical neurons following OGD

**Fig. 1:**
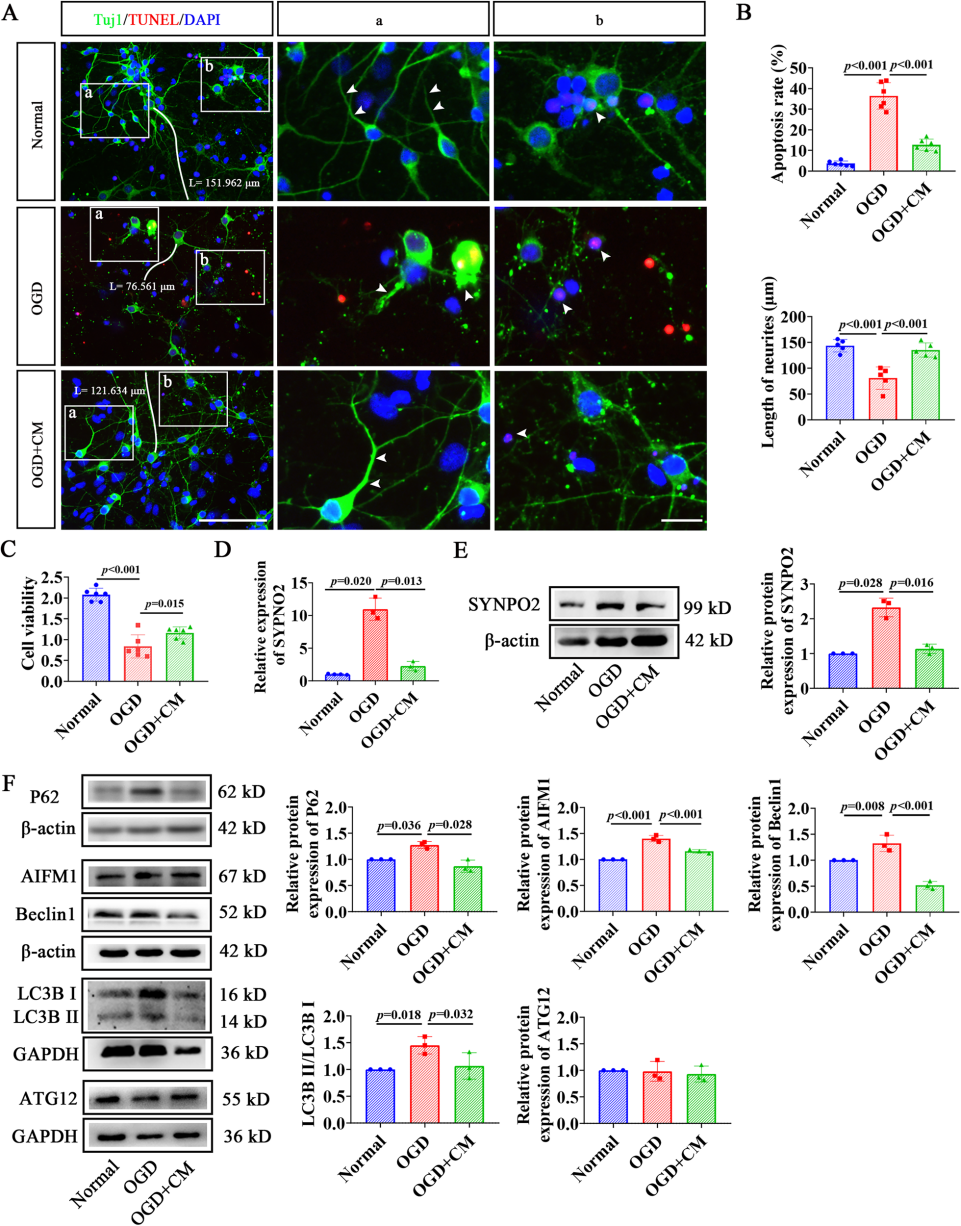
CM suppressed neuronal autophagic injury in OGD neurons. **A** Immunofluorescence double staining of cortical neurons with Tuj1 (green) and TUNEL (red) in the Normal, OGD, OGD + CM groups. Scale bar = 100 μm. Blue, DAPI-stained nuclei. **a** Magnified view of neuronal axons indicated by white arrows. **b** Magnified view of apoptotic neurons indicated by white arrows. Scale bar = 25 μm (**a**, **b**). **B** Quantitative bar graph of apoptotic rate and neuronal axon length in the Normal, OGD, OGD + CM groups. **C** Quantitative bar graph showing cell viability in neurons from Normal, OGD, OGD + CM groups. **D** RT-qPCR detected relative expression of SYNPO2 in neurons 24 hours after OGD. **E**. WB detected expression of SYNPO2 in neurons 24 hours after OGD. **F**. Expression of autophagy-related proteins, P62, AIFM1, Beclin-1, LC3B, and ATG12 in the Normal, OGD, and OGD + CM groups. Bar graphs display mean ± SD of independent biological replicates. *n* = 3–6 wells, two-sided unpaired one-way ANOVA. All experiments were repeated three times independently, with similar results. OGD oxygen-glucose deprivation; CM BMSC-conditioned medium.

To assess the effects of BMSCs on neuroautophagy, we induced OGD in cultured cortical neurons and administered CM immediately after OGD, maintaining the treatment for 24 hours. Immunofluorescence and quantitative analysis revealed that apoptosis and axonal injury were significantly increased in the OGD group compared to the Normal group with a marked reduction in average axon length (151.962 μm vs. 76.561 μm, Fig. 1A, B, *p* < 0.001). However, after CM intervention, the length of OGD-injured neuronal axons was significantly prolonged (76.561 μm vs. 121.634 μm, *p* < 0.001) and neuronal apoptosis were prominently reduced (Fig. 1A, B, *p* < 0.001). Cell viability deceased significantly at 24 hours post-OGD (Fig. 1C, *p* < 0.001), but was improved in the CM group compared to the OGD group (Fig. 1C, *p* = 0.015). Additionally, RT-qPCR and WB detected a significant increase in both mRNA and protein levels of SYNPO2 in the OGD group in comparison to the Normal group (*p* = 020, *p* = 0.028), which were notably declined after CM intervention (Fig. S1, Fig. 1D, E, *p* = 0.013, *p* = 0.016). WB analysis also showed that the expression of key autophagy-related proteins, P62, AIFM1, Beclin1, and ratio of LC3B II/I were markedly upregulated in the OGD group (Fig. S1, Fig. 1F, *p* = 0.036, *p* < 0.001, *p* = 0.008, *p* = 0.018), but were significantly downregulated in the CM group (Fig. S1, Fig. 1F, *p* = 0.028, *p* < 0.001, *p* < 0.001, *p* = 0.032).

### BMSCs attenuated autophagy to improve neurological dysfunction in HIE rats

**Fig. 2:**
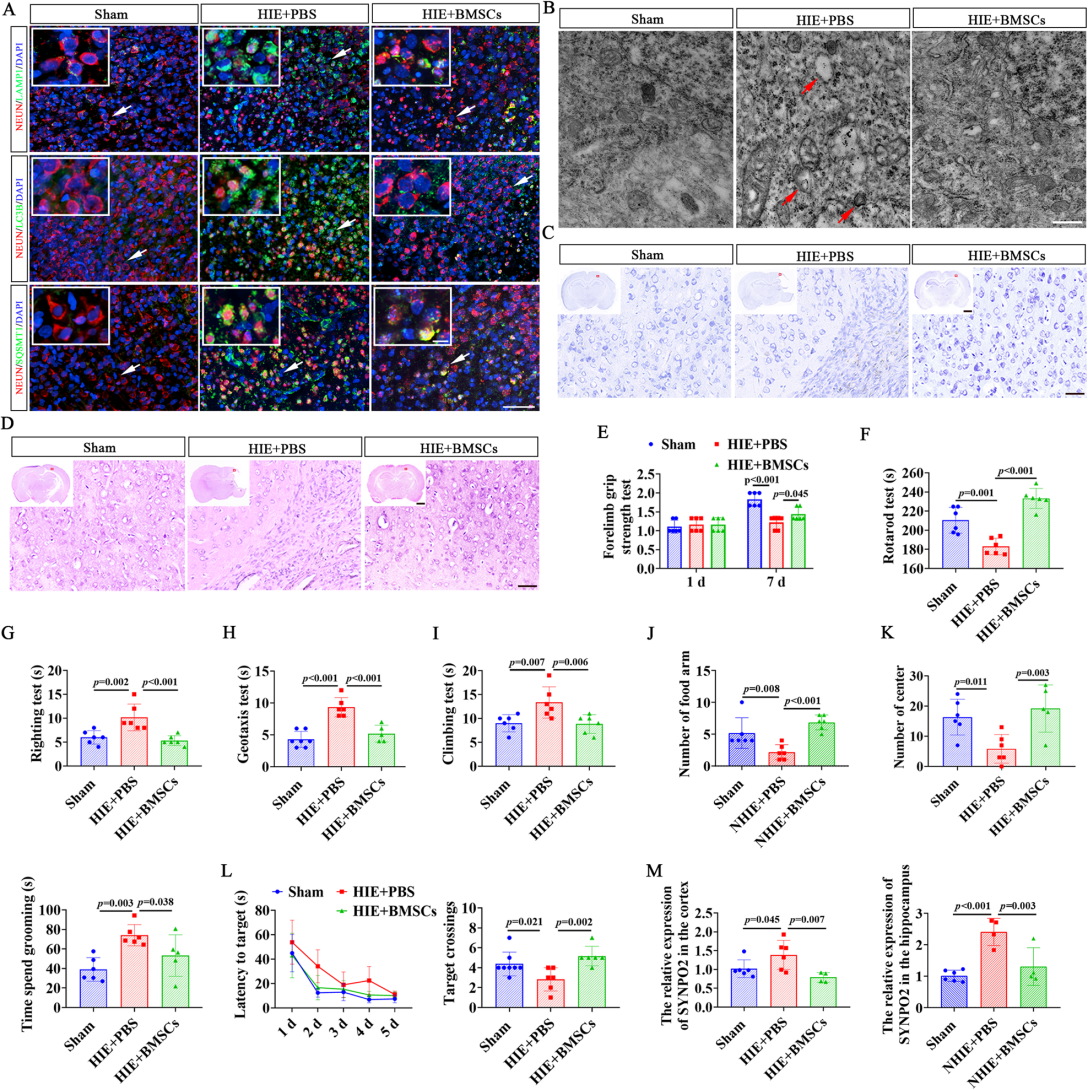
BMSCs alleviated brain autophagic injury and neurological dysfunction in HIE rats. **A** Immunofluorescent double-labeling staining of brain tissues with NEUN (red) and autophagic markers (green) in HIE rats. Scale bar = 50 μm, 25 μm (magnified view). Blue, DAPI-stained nuclei. **B** TEM images showing autophagy in the brain tissue of HIE rats, with red arrows indicating autophagic vesicles. Scale bar = 500 nm. Nissl staining (**C**) and HE staining (**D**) of coronal brain sections from HIE rats in the Sham, HIE + PBS, HIE+BMSCs groups. Scale bar = 2 mm (panoramic view), 500 μm (magnified view). Forelimb grip test (**E**), rotarod test (**F**), righting test (**G**), geotaxis test (**H**), and climbing test (**I**) were used to evaluate the muscle strength and coordination in HIE rats. **J** Spatial exploration and autonomous behavior of HIE rats were detected by the Y Maze. **K** The number of entries into the center of the open field (left) and grooming time (right). **L** The latency to target and the number of platform crossings in the Sham, HIE, HIE+BMSCs groups. **M** RT-qPCR verified the expression of SYNPO2 in the Sham, HIE + PBS, HIE+BMSCs groups. Bar graphs display mean ± SD of independent biological replicates. *n* = 4-8 rats, two-sided unpaired one-way ANOVA. All experiments were repeated three times independently, with similar results. HIE + PBS, rats were given PBS before HIE modeling as control; HIE+BMSC, rats were infused with BMSCs before HIE modeling; TEM transmission electron microscope.

We next investigated the role of BMSCs (2 × 10^5^/5 μL) in regulating autophagy in cerebral neurons using HIE rats. The results of immunofluorescence staining and TEM showed that the autophagy and its markers (LAMP1, LC3B, and SQSMT1) were significantly increased in HIE rats, accompanied by neuronal damage in the brain tissues of HIE rats (Fig. 2A, B). In contrast, HIE rats transplanted with BMSCs showed an obvious decrease in autophagy markers and evidence of neuronal repair (Fig. 2A, B). The protective effects of BMSCs on neurons was further assessed by HE staining and Nissl staining, which showed that tissue defects, disordered cell arrangement, and widespread neuronal death were present in the HIE group (Fig. 2C, D). Whereas, BMSC-treated HIE rats exhibited a significant increase in the number of normal neurons and Nissl bodies, as well as improvement in the right hemisphere defects (Fig. 2C, D). To evaluate functional recovery, we performed several behavioral tests, including forelimb grip test, rotarod test, righting test, geotaxis test, and climbing test. We found that compared to Sham group, neonatal HIE rats presented significant impairments in grip strength and rotarod performance (Fig. 2E, F, *p* < 0.001, *p* = 0.001), as well as delayed recovery time in the righting, geotaxis, and climbing tests, indicated by the prolonged time they were taken to reverse 180° upward and climb to the set line when compared to the Sham group (Fig. 2G-I, *p* = 0.002, *p* < 0.001, *p* = 0.007). However, BMSC treatment significantly improved strength and coordination deficits (Fig. 2E-I, *p* = 0.045, *p* < 0.001, *p* < 0.001, *p* < 0.001, *p* = 0.006). To evaluate cognitive functions, we conducted open field, Y-maze, and water maze experiments. HIE rats exhibited reduced spatial exploration and increased grooming behavior (Fig. 2J, K, *p* = 0.008, *p* = 0.011, *p* = 0.003, Fig. S2A, B), but BMSC treatment significantly enhanced exploratory activity and depressed anxious behavior (Fig. 2J, K, *p* < 0.001, *p* = 0.003, *p* = 0.038, Fig. S2A, B). Similarly, the water maze test revealed impaired spatial memory in HIE rats, as evidenced by a significant reduction in the number of rats crossing the platform (Fig. 2L, *p* = 0.021, Fig. S2C), which was markedly improved after BMSC treatment (Fig. 2L, *p* = 0.002, Fig. S2C). Additional RT-qPCR detection revealed that SYNPO2 was highly expressed in the cortical and hippocampus of HIE rats (Fig. 2M, *p* = 0.045, *p* < 0.001), but significantly decreased in the BMSC group (Fig. 2M, *p* = **0.007,** *p* = **0.003**), indicating that SYNPO2 was specifically expressed in the CNS after HIE. These results demonstrated that BMSC treatment significantly attenuated autophagy-related dysfunction, suppressed SYNPO2 expression, and enhanced neuronal survival and functional recovery following HIE injury. The neuroprotective effects of BMSCs appear to be closely linked to their ability to modulate autophagy and mitigate neuronal damage.

### Interference with SYNPO2 ameliorates autophagic damage in OGD neurons

**Fig. 3:**
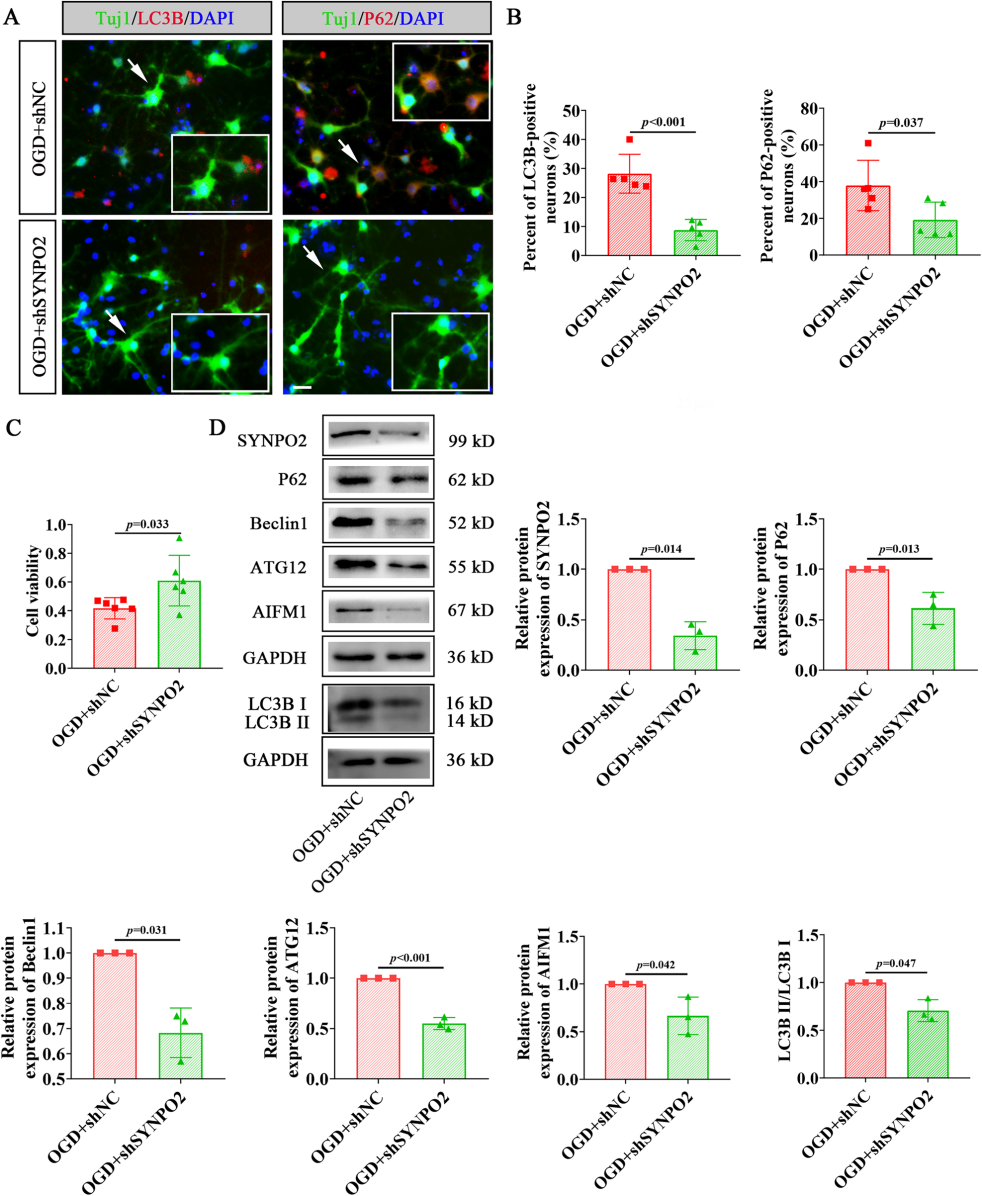
Changes of autophagy-related proteins in cortical neurons infected with shSYNPO2 lentivirus. **A** Immunofluorescent staining of cortical neurons with Tuj1 (green)/LC3B (red) and Tuj1 (green)/P62 (red) in OGD+shNC and OGD+shSYNPO2 groups. Scale bar = 25 μm. **B** Quantification of LC3B and P62-positive neurons from immunofluorescent staining. **C** Cell viability of neurons in OGD+shNC and OGD+shSYNPO2 groups. **D** WB detection of SYNPO2 and autophagy-related protein expression (P62, Beclin1, ATG12, AIFM1 and LC3B II/I) in the OGD+shNC and OGD+shSYNPO2 groups. Bar graphs display mean ± SD of independent biological replicates. *n* = 3–6 wells, two-sided unpaired independent sample *t*-test. All experiments were repeated three times independently, with similar results. OGD+shNC, cortical neurons infected with control lentivirus overexpressing SYNPO2 before OGD; OGD+shSYNPO2, cortical neurons infected with shSYNPO2 lentivirus before OGD.

To explore the role of SYNPO2 in neuronal autophagy, primary cortical neurons were infected with shNC and shSYNPO2 lentivirus in vitro (Fig. S3). Immunofluorescence staining revealed that autophagy-related markers LC3B and P62 were significantly elevated in cortical neurons 24 hours after OGD but were reduced following SYNPO2 interference (Fig. 3A, B, *p* < 0.001, *p* = 0.037). Besides, neuronal viability assessed by CCK8 assay showed a significant increase in the shSYNPO2 group compared to shNC group after OGD (Fig. 3C, *p* = 0.033). In addition, WB analysis demonstrated the expression of autophagy-related proteins, P62, Beclin1, ATG12, AIFM1 and ratio of LC3B II/I, were significantly decreased in OGD neurons with shSYNPO2 interference (Fig. S1, Fig. 3D, *p* = 0.014, *p* = 0.013, *p* = 0.031, *p* < 0.001, *p* = 0.042, *p* = 0.047). These data suggest that interference with SYNPO2 inhibits the activation of autophagy and alleviates neuronal damage induced by OGD.

### Interference with SYNPO2 attenuated neuronal autophagy and improved neurological dysfunction in HIE rats

**Fig. 4:**
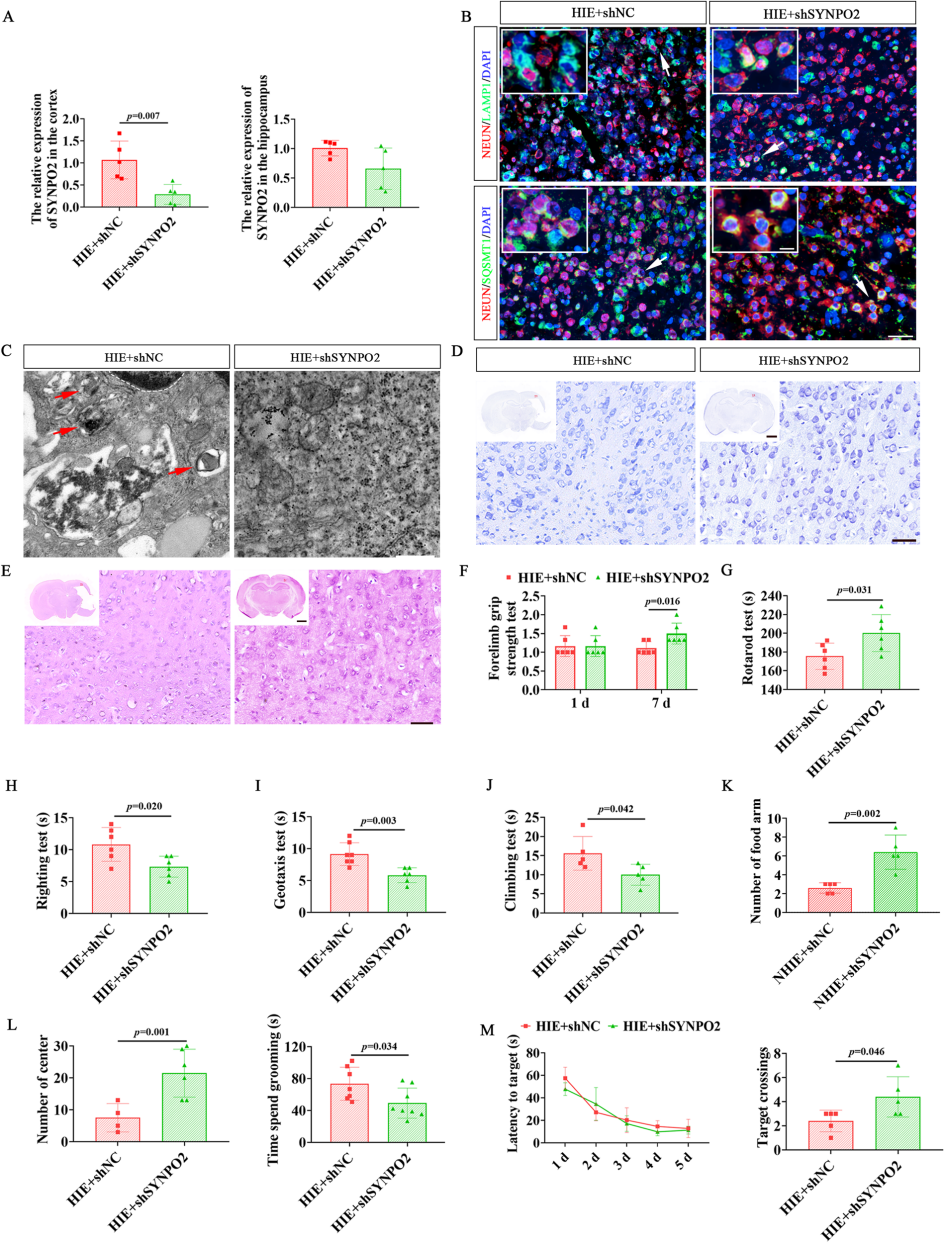
Suppression of SYNPO2 in HIE rats alleviated neuroautophagic damage and neurological dysfunction. **A** RT-qPCR results showing the expression levels of SYNPO2 in the cortex and hippocampus of HIE rats. **B** Immunofluorescent double-labeling staining of brain tissues of HIE rats with NEUN (red) and autophagic markers (green). Scale bar = 50 μm, 25 μm (magnified view). Blue, DAPI-stained nuclei. **C** TEM images showing autophagy in the brain tissue of HIE rats, with red arrows indicating autophagic vesicles. Scale bar = 500 nm. Nissl staining (**D**) and HE staining (**E**) of coronal brain sections from HIE rats in the HIE+shNC and HIE+shSYNPO2 groups. Scale bar = 2 mm (panoramic view), 500 μm (magnified view). Evaluation of muscle strength and coordination ability of rats by forelimb grip test (**F**), rotarod test (**G**), righting test (**H**), geotaxis test (**I**), and climbing test (**J**). The spatial exploration, autonomous behavior, and anxiety performance in HIE rats were detected by the Y maze (**K**) and open field test (**L**). **M**. The latency to target and the number of platform crossings in the HIE+shNC and HIE+shSYNPO2 groups. Bar graphs display mean ± SD of independent biological replicates. *n* = 4–8 rats, two-sided unpaired independent sample *t*-test. All experiments were repeated three times independently, with similar results. HIE+shNC, rats injected with control interfering lentivirus before HIE modeling; HIE+shSYNPO2, rats injected with SYNPO2 interfering lentivirus before HIE modeling; TEM, Transmission electron microscope.

To explore the role of SYNPO2 in HIE, rats on postnatal day 3 were injected with shSYNPO2 and shNC lentivirus into the lateral ventricle. Four days later, HIE models were constructed to investigate the potential relationship between SYNPO2 and HIE pathology. Firstly, RT-qPCR detection confirmed significantly lower SYNPO2 expression in cerebral cortex of HIE rats after SYNPO2 interference (Fig. 4A, *p* = **0.007**). Immunofluorescence staining showed reduced expression of neuronal autophagy-related molecules in HIE rats treated with shSYNPO2 compared to shNC group (Fig. 4B). TEM assay further demonstrated increased autophagy (red arrowheads), destroyed cell structure and disrupted nucleolus boundary (white dotted lines) in the brain cortex of HIE rats in shNC group, while SYNPO2 interference alleviated these autophagic and structural damages (Fig. 4C). Nissl and HE staining exhibited that compared with the shNC group, the HIE rats in the shSYNPO2 group had preserved brain tissue integrity, increased Nissl-positive neurons, and restored cell structure (Fig. 4D, E).

Behavioral tests highlighted improved neurological function in shSYNPO2-treated rats. Grip strength and rotarod performance time were markedly extended, while righting test time was shortened compared to shNC-treated rats (Fig. 4F-H, *p* = 0.016, *p* = 0.031, *p* = 0.020). Similarly, geotaxis and climbing test time was significantly reduced in comparison with shNC rats, indicating restored muscle strength and coordination in shSYNPO2-treated HIE rats (Fig. 4I, J, *p* = 0.003, *p* = 0.042). Additionally, shSYNPO2 HIE rats exhibited more entries into the food arm of the Y-maze, augmented time spent in the center of the open field, and decreased platform crossings in the water maze test, in comparison with shNC group (Fig. 4K-M, Fig. S4, *p* = 0.002, *p* = 0.001, *p* = 0.034, *p* = 0.046), indicating exploratory behavior was significantly enhanced, and anxious behavior was effectively alleviated. These outcomes indicated that SYNPO2 is involved in the regulation of neuroautophagy injury and neurological dysfunction in HIE rats. Suppressing SYNPO2 expression effectively alleviates autophagy-related neuronal injury and improves behavioral outcomes.

### CM ameliorated autophagy impairment in OGD neurons by downregulating SYNPO2 expression

**Fig. 5:**
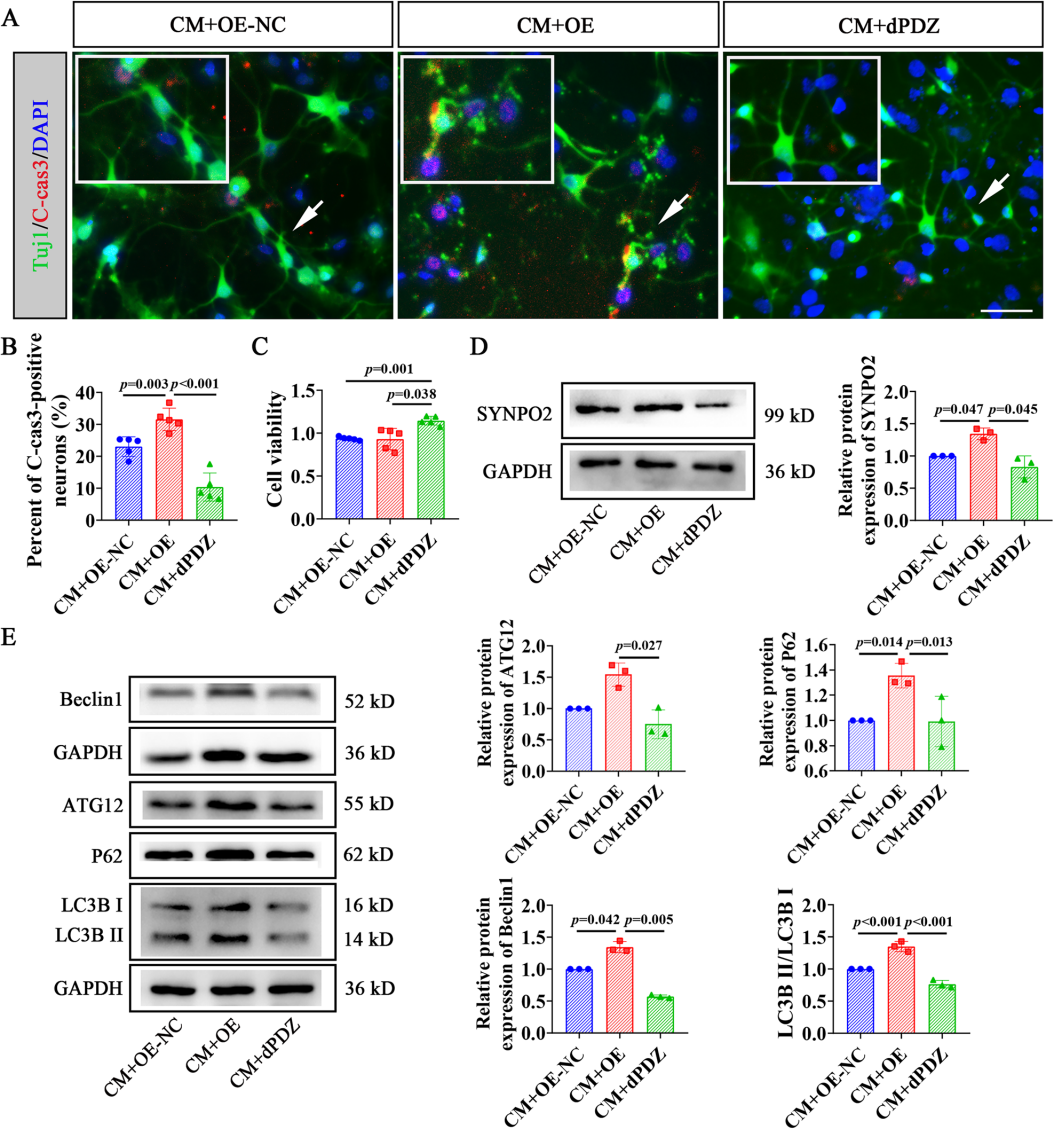
CM reduced OGD-induced neuronal damage and autophagy by downregulating SYNPO2 expression. **A**. Immunofluorescence staining of cortical neurons with TUJ1 (green) and C-cas3 (red) in the CM + OE-NC, CM + OE and CM+dPDZ groups. Scale bar = 50 μm. White arrows indicate apoptotic neurons. Blue, DAPI-stained nuclei. **B** Quantification of C-cas3-positive neurons from CM + OE-NC, CM + OE and CM+dPDZ groups. **C** Cell viability of neurons in CM + OE-NC, CM + OE and CM+dPDZ groups. **D** WB detection of SYNPO2 expression in the OGD+shNC and OGD+shSYNPO2 groups. **E** WB detection of autophagy-related protein expression (ATG12, P62, Beclin1 and LC3B II/I) in the neurons from CM + OE-NC, CM + OE and CM+dPDZ groups. Bar graphs display mean ± SD of independent biological replicates. *n* = 3–5 wells, two-sided unpaired one-way ANOVA. All experiments were repeated three times independently, with similar results. dPDZ, cortical neurons infected with the SYNPO2-dPDZ deletion mutant lentivirus before OGD and treated with BMSC-conditioned medium; CM + OE-NC, cortical neurons infected with SYNPO2 overexpressing control lentivirus before OGD and subjected to BMSC treatment; CM + OE, cortical neurons infected with SYNPO2 overexpressing lentivirus before OGD and with BMSC infusion.

To further explore whether BMSCs regulate neuroautophagy through SYNPO2, we infected primary cortical neurons with lentiviruses overexpressing SYNPO2 (OE), overexpression SYNPO2-NC (OE-NC) and SYNPO2-dPDZ deletion mutant (dPDZ) (Fig. S5). CM was added immediately after neuronal OGD and maintained for 24 hours. Immunofluorescence staining showed increased number of C-cas3-positive neurons, and axonal rupture in OGD neurons overexpressing SYNPO2 (Fig. 5A, B, *p* = 0.003). In contrast, these neuronal injuries were significantly rescued in OGD neurons with dPDZ mutation (Fig. 5A, B, *p* < 0.001). Neuronal viability increased significantly in CM+dPDZ group compared to CM + OE-NC and CM + OE groups (Fig. 5C, *p* < 0.001, *p* = 0.038), indicating that SYNPO2 deletion enhanced the beneficial effects of CM on damaged neurons. Additionally, WB confirmed a significant increase in protein levels of SYNPO2 in the CM + OE group in comparison to the NC group (*p* < 0.047), which were notably declined in CM+dPDZ group (Fig. 5D, Fig. S1, *p* = 0.045). In the WB outcomes, the expression of P62, Beclin1, and ratio of LC3B II/I in OGD neurons overexpressing SYNPO2 was markedly elevated with addition of CM (Fig. 5E, Fig. S1, *p* = 0.014, *p* = 0.042, *p* < 0.001), while autophagic activation was significantly inhibited in OGD neurons with SYNPO2-dPDZ mutation after CM intervention, indicated by the prominent decrease of ATG12, P62, Beclin1 and ratio of LC3B II/I (Fig. 5E, Fig. S1, *p* = 0.027, *p* = 0.013, *p* = 0.005, *p* < 0.001). These findings confirmed that CM could alleviate autophagy-induced neuronal damage by downregulating SYNPO2 expression.

### BMSCs downregulated SYNPO2 to alleviate neuroautophagy and improve dysfunction in HIE rats

**Fig. 6:**
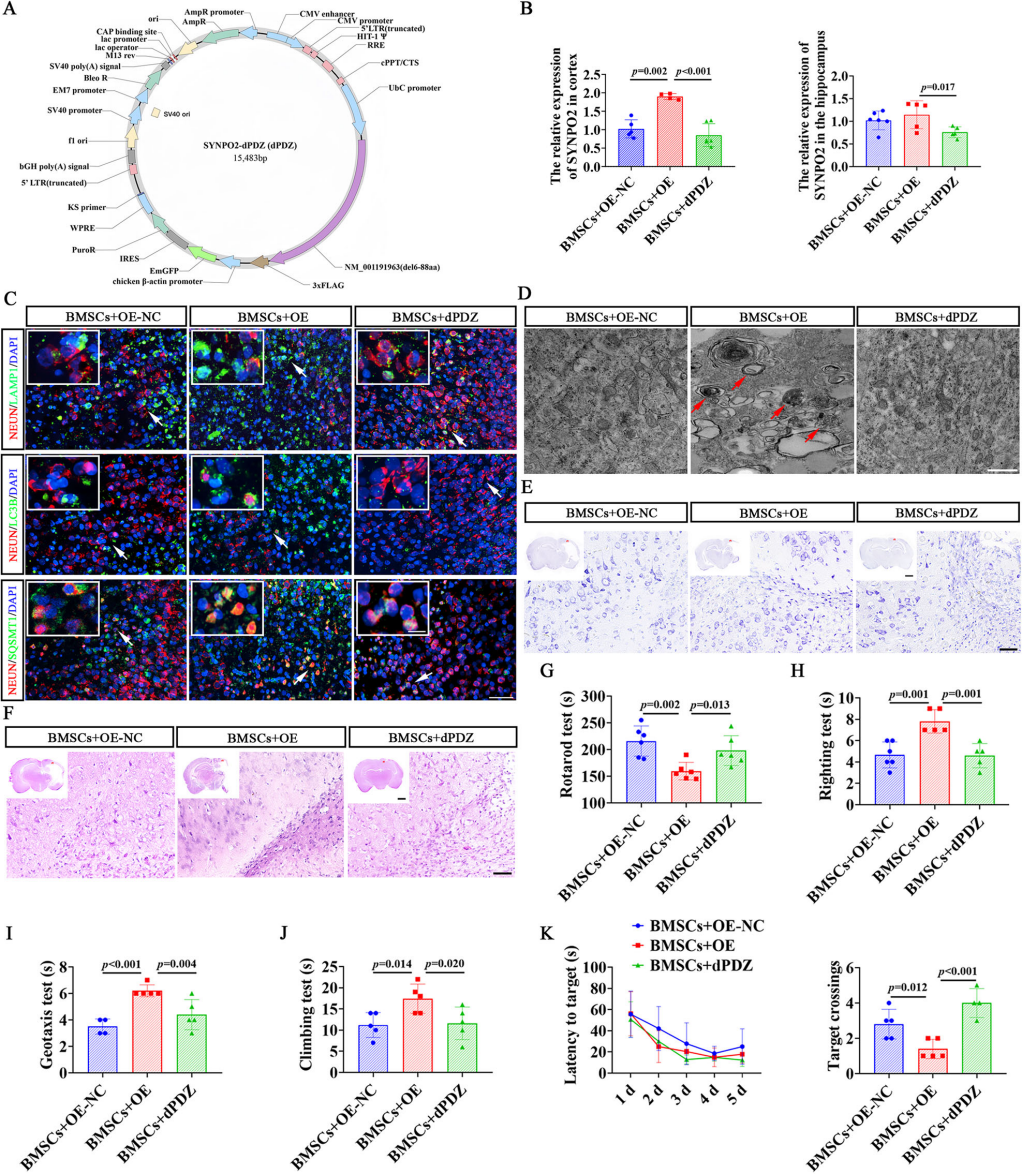
SYNPO2 downregulation by BMSC treatment ameliorated autophagy and neurological dysfunction in HIE rats. **A** Lentiviral vector constructs of SYNPO2-dPDZ used in this study. **B** RT-qPCR results showing the relative expression levels of SYNPO2 in the cortex and hippocampus of HIE rats. **C** Immunofluorescent double-labeling staining of brain tissues of HIE rats with NEUN (red) and autophagic markers (green) in HIE rats. Scale bar = 50 μm, 25 μm (magnified view). Blue, DAPI-stained nuclei. **D** TEM images showing autophagy in the brain tissue of HIE rats, with red arrows indicating autophagic vesicles. Scale bar = 500 nm. Nissl staining (**E**) and HE staining (**F**) of coronal brain sections from HIE rats in the BMSCs+OE-NC, BMSCs+OE, BMSCs+dPDZ groups. Scale bar = 2 mm (panoramic view), 500 μm (magnified view). The muscle strength and coordination ability of rats in the BMSCs+OE-NC, BMSCs+OE, BMSCs+dPDZ groups were tested by rotarod test (**G**), righting test (**H**), geotaxis test (**I**), and climbing test (**J**). **K** The latency to target and the number of platform crossings in water maze test. Bar graphs display mean ± SD of independent biological replicates. *n* = 4-6 rats, two-sided unpaired one-way ANOVA. All experiments were repeated three times independently, with similar results. BMSCs+OE-NC, rats injected with SYNPO2 overexpressing control lentivirus before HIE model establishment and BMSCs treatment; BMSCs+OE, rats injected with SYNPO2 overexpressing lentivirus before HIE model establishment and BMSCs treatment; BMSCs+dPDZ, rats injected with SYNPO2-dPDZ deletion mutant lentivirus before HIE model establishment and BMSCs treatment; TEM transmission electron microscope.

We next validated the role of SYNPO2 in the neuroprotective effects of BMSCs by transfecting HIE rats with SYNPO2 overexpression and depletion mutant lentivirus (Fig. 6A). RT-qPCR detection identified an increase of SYNPO2 expression in the cortex of HIE rats in SYNPO2-OE group (Fig. 6B, *p* = 0.002), and decreased expression in the cortex and hippocampus of SYNPO2-dPDZ group (Fig. 6B, *p* < 0.001, *p* = 0.017). One day after HIE induction, rats were subjected to multiple immunofluorescence staining and TEM. The results showed enhanced levels of SQSTM1, LAPM1, LC3B, and obvious autophagic vesicles (red arrow), along with neuronal damage in the brain tissue of rats with SYNPO2 overexpression (Fig. 6C, D). Whereas, in the SYNPO2-dPDZ group, BMSC treatment reduced expression of autophagy markers and alleviated neuronal damage (Fig. 6C, D). Meanwhile, Nissl and HE staining results confirmed that rats in the SYNPO2-dPDZ group with BMSC treatment exhibited less disruption in tissue integrity disruption and neuronal damage compared to SYNPO2-OE rats with BMSC treatment(Fig. 6E, F).

In addition, behavioral tests revealed that SYNPO2 overexpression impaired muscle strength and coordination in BMSC-treated HIE rats compared to the OE-NC group (Fig. 6G–J, *p* = 0.002, *p* = 0.001, *p* < 0.001, *p* = 0.014), whereas rats in the SYNPO2-dPDZ group exhibited improved neurological function in HIE rats with BMSC treatment (Fig. 6G-J, *p* = 0.013, *p* = 0.001, *p* = 0.004, *p* = 0.020). These results indicated that SYNPO2 overexpression reversed the ameliorative effects of BMSCs on neurological dysfunction in HIE rats, whereas SYNPO2 deletion restored the therapeutic effects of BMSCs. Similarly, in the Morris water maze assay, BMSC-treated HIE rats with SYNPO2 overexpression exhibited reduced number of platform crossings and impaired memory in compared to OE-NC group (Fig. 6K, Fig. S6, *p* = 0.012). However, in SYNPO2-dPDZ HIE rats, learning and memory impairments were significantly alleviated with BMSC treatment (Fig. 6K, Fig. S6, *p* < 0.001). These results suggested that BMSCs might regulate SYNPO2 expression to depress autophagy, thereby ameliorating neurological damage and dysfunction in HIE rats.

## Discussion

In this study, we observed increased SYNPO2 expression, and heightened autophagy and neuroautophagy impairment in OGD neurons and HIE rats. Treatment with BMSCs attenuated autophagic injury and neuronal dysfunction both in vitro and in vivo. Similarly, interference with SYNPO2 reduced autophagy and mitigated neurological damage, suggesting that SYNPO2 plays a critical role in regulating neuroautophagic damage following HIE. Furthermore, overexpression of SYNPO2 depressed the neuroprotective effects of BMSCs on HIE-induced autophagic injury, while neuroprotective efficacy of BMSCs were retained when SYNPO2-dPDZ deletion mutation were applied. These findings demonstrate that SYNPO2 contributes to autophagic injury induced by HIE. By downregulating SYNPO2 expression, BMSCs effectively reduce autophagy, thereby improving neuronal autophagic injury and dysfunction.

### BMSCs prevent autophagy and improve neurological deficiency

HIE is a leading cause of neonatal disability and mortality, with an urgent need for novel treatment strategies and an improved understanding of underlying mechanisms [46, 47]. Preclinical studies can improve outcomes in HIE through mechanisms such as reducing inflammation, promoting neurogenesis, and repairing neural damage [17, 48]. In recent years, studies have found that stem cells play an important role in maintaining cell survival and attenuating cell damage by regulating autophagy [49–51]. For instance, MSCs have been shown to suppress excessive autophagy and alleviate tissue damage in various conditions, including myocardial ischemia and ischemic stroke [52]. Similarly, human umbilical cord mesenchymal stem cells (hUC-MSCs) alleviate excessive autophagy of ovarian granulosa cells and improve ovarian function through the VEGFA/PI3K/AKT/mTOR pathway [53]. In our study, OGD neurons exhibited increased autophagy-related proteins, axonal rupture, and reduced cell viability. BMSC-CM intervention reduced axonal damage, diminished neuro autophagic injury, and improved cell viability. Similarly in previous studies, adipose-derived stem cells (ADSCs) reduced autophagy in stroke mice, thereby inducing neuroprotection effects [54]. MSCs transplantation inhibited autophagy in hippocampal neurons of HIE rats and effectively improves their memory function [55]. Autophagy and neurological damage are blocked and neurological recovery is promoted in ischemic stroke animals after MSCs treatment [56]. In our HIE model, neuroautophagy impairment and elevated cortical hippocampal SYNPO2 expression were associated with deficits in muscle strength, coordination, and memory. BMSC intervention reduced autophagy, downregulated SYNPO2 expression, and mitigated these neurological dysfunctions. These findings align with prior research, further supporting the neuroprotective effects of BMSCs via autophagy regulation. Our study provides new evidence for the therapeutic potential of BMSCs in HIE and highlights SYNPO2 as a key target in this process.

### SYNPO2 regulated neuroautophagy damage

SYNPO2 is a structural protein ubiquitously expressed in muscle tissues, where it plays roles in microfilament assembly, cancer progression, and autophagy regulation [57, 58]. Chaperone-assisted selective autophagy (CASA), in which SYNPO2 interacts with BAG3, was identified as a tension-induced autophagy pathway [36]. The autophagosome-promoting function of SYNPO2 relies on its PDZ structural domain, which recruits the autophagy ubiquitin receptor SQSTM1. In this process, molecular chaperones such as Hsc70, HspB8, and BAG3 form a CASA complex, and the WW domain of BAG3 interacts with the PDZ domain of SYNPO2 to promote autophagosome membrane fusion, triggering autophagy [31, 37]. Under cellular stress, the expression of SYNPO2 is upregulated, and its PDZ motif binds to autophagy-related proteins, thereby affecting the occurrence of CASA [59, 60]. Besides, SYNPO2 contributes to protein quality control by degrading unfolded or damaged proteins via autophagy, while its deficiency disrupts myofiber stability and autophagy regulation in muscle tissues [31, 61]. However, its role in neuronal autophagic damage remains underexplored. Our study revealed that interfering with SYNPO2 attenuated autophagy, ameliorated neuronal damage, enhanced neuronal activity, and repaired neurological dysfunction in HIE rats. This aligns with findings on SYNPO, a homolog of SYNPO2, which is involved in the structural and functional plasticity of dendritic spines and plays a crucial role in the ability of neurons to undergo a long-term calcium storage-related capacity [32, 62, 63]. Moreover, SYNPO affects autophagic flux in mature neurons by interacting with BAG3 and inhibits autophagosome membrane extension when interfering with SYNPO expression [35, 38]. These observations, together with our results, suggest that SYNPO2 is a key regulator of autophagic apoptosis in neurons following neonatal HIE.

### BMSCs down-regulated SYNPO2 to reduce autophagy and alleviated neurological dysfunction in HIE rats

BMSCs are well-documented to ameliorate ischemic injury in different tissues. In acute myocardial infarction induced by ischemia and hypoxia, BMSCs inhibit autophagy through mTOR signaling pathway to improve myocardial damage. Mechanistically, extracellular vesicles from BMSCs deliver miR-144-3p to ischemic and hypoxic cardiomyocytes, targeting ROCK1 and activate the PI3K/AKT/mTOR pathway to inhibit ischemia and hypoxia-induced apoptosis and autophagy in cardiomyocytes [64]. Similarly, BMSC-CM protects H9c2 cardiomyocytes from hypoxia/reoxygenation-induced injury by modulating notch2/mTOR/autophagy signaling pathway [65], while BMSC-derived exosomal miRNA-29c attenuates cardiac ischemia/reperfusion injury by inhibiting excessive autophagy via PTEN/Akt/mTOR signaling pathway [66]. Although the molecular mechanisms by which BMSCs modulate ischemia-hypoxia-induced neuroautophagic injury remain to be elucidated, studies have shown that MSC transplantation reduces p-AMPK expression in hippocampal neurons, decreases autophagy via the AMPK/mTOR pathway, and improves memory function in HIBD rats [28]. Recent evidence also highlights SYNPO2 as a key regulator of autophagy in different cellular processes [52, 60]. In our study, we found that BMSC treatment down-regulated SYNPO2 expression, reduced apoptotic neurons, suppressed expression levels of autophagy markers (ATG12, P62, Beclin1, LC3B), and improved neurological dysfunction in HIE rats. Meanwhile, interference with SYNPO2 produced neuroprotective effects similar to BMSC treatment. Further investigations demonstrated that overexpression of SYNPO2 reversed the beneficial effects of BMSCs, while SYNPO2-dPDZ deletion mutations preserved the neuroprotective efficacy of BMSCs. These findings suggest that SYNPO2 is involved in neuroautophagy regulation, and its down-regulation by BMSCs represents a novel mechanism to ameliorate neuroautophagic damage and neurological dysfunction in HIE.

## Conclusions

Our study demonstrated that SYNPO2 plays a critical role in neuronal autophagy following neonatal HIE and that BMSCs can modulate autophagy via SYNPO2 to alleviate HIE symptoms. These findings highlight SYNPO2 as a potential therapeutic target and provide a novel mechanism for stem cell-based treatments for neonatal HIE. This work paves the way for the development of more precise therapeutic strategies targeting neuronal autophagy in neonatal HIE.

## Supplementary information

Supplemental figures and legends

Original data for WB

Original data for qPCR
