# Supplementary material for: Bone marrow mesenchymal stem cells alleviate neurological dysfunction by reducing autophagy damage via downregulation of SYNPO2 in neonatal hypoxic–ischemic encephalopathy rats
Source: Cell Death Dis. 2025 Feb 25;16(1):131. doi: 10.1038/s41419-025-07439-w (PMC11862179; doi:10.1038/s41419-025-07439-w)
Supplement: Supplementary file 1 — Supplemental figures and legends [file 41419_2025_7439_MOESM1_ESM.docx]

**
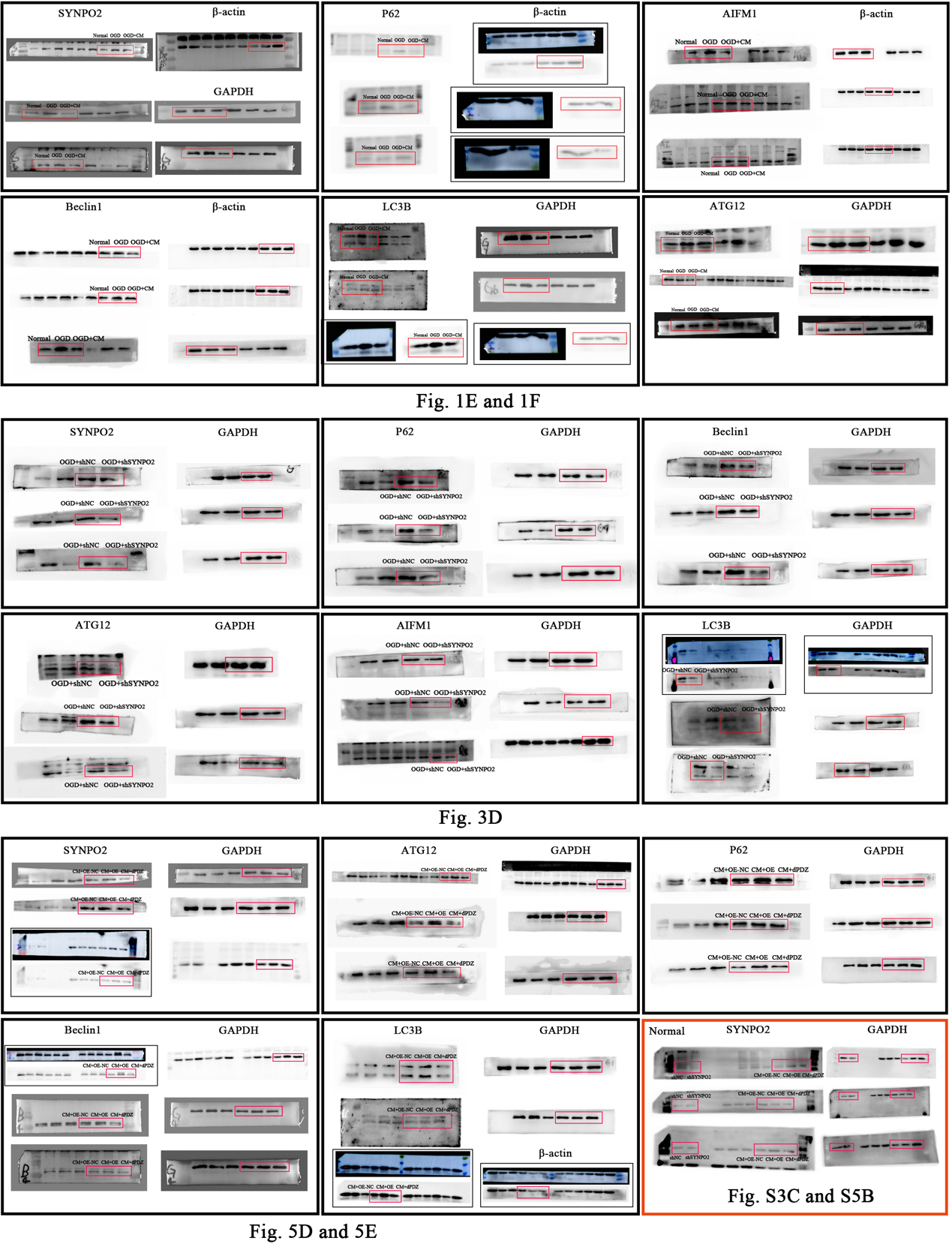
**

**Fig. S1 Original protein bands for WB experiments. Related to Fig. 1, Fig. 3 and Fig. 5.**

**
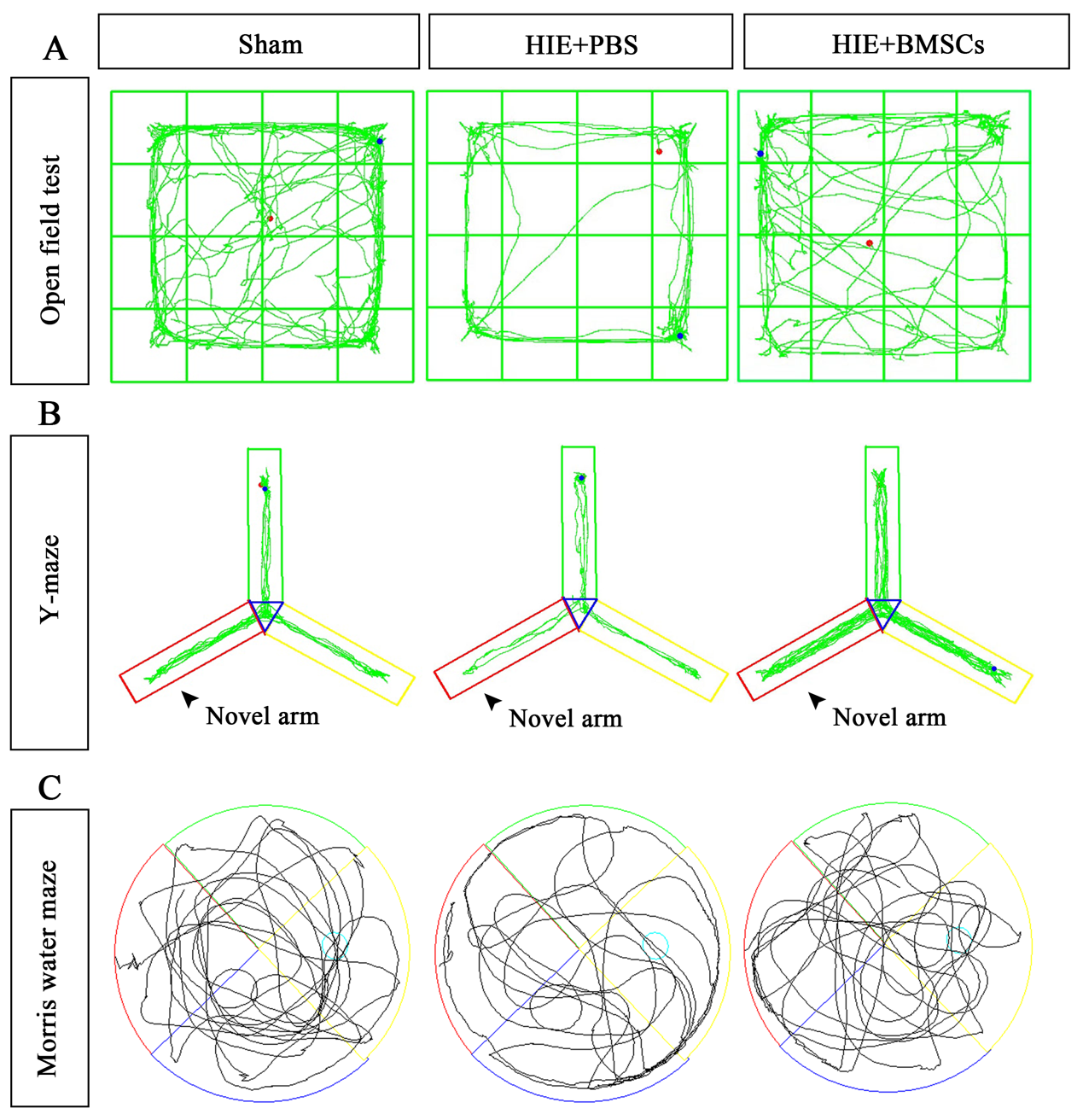
**

**Fig. S2 Autonomous exploratory ability and memory function in HIE rats rescued by BMSCs. A-C.** Trajectories of rats in the open field **(A)**, Y-maze **(B),** and Morris water maze **(C)** experiments. n = 4-8 rats. HIE+PBS, rats were given PBS before HIE modeling as control; HIE+BMSCs, rats were infused with BMSCs before HIE modeling.

**
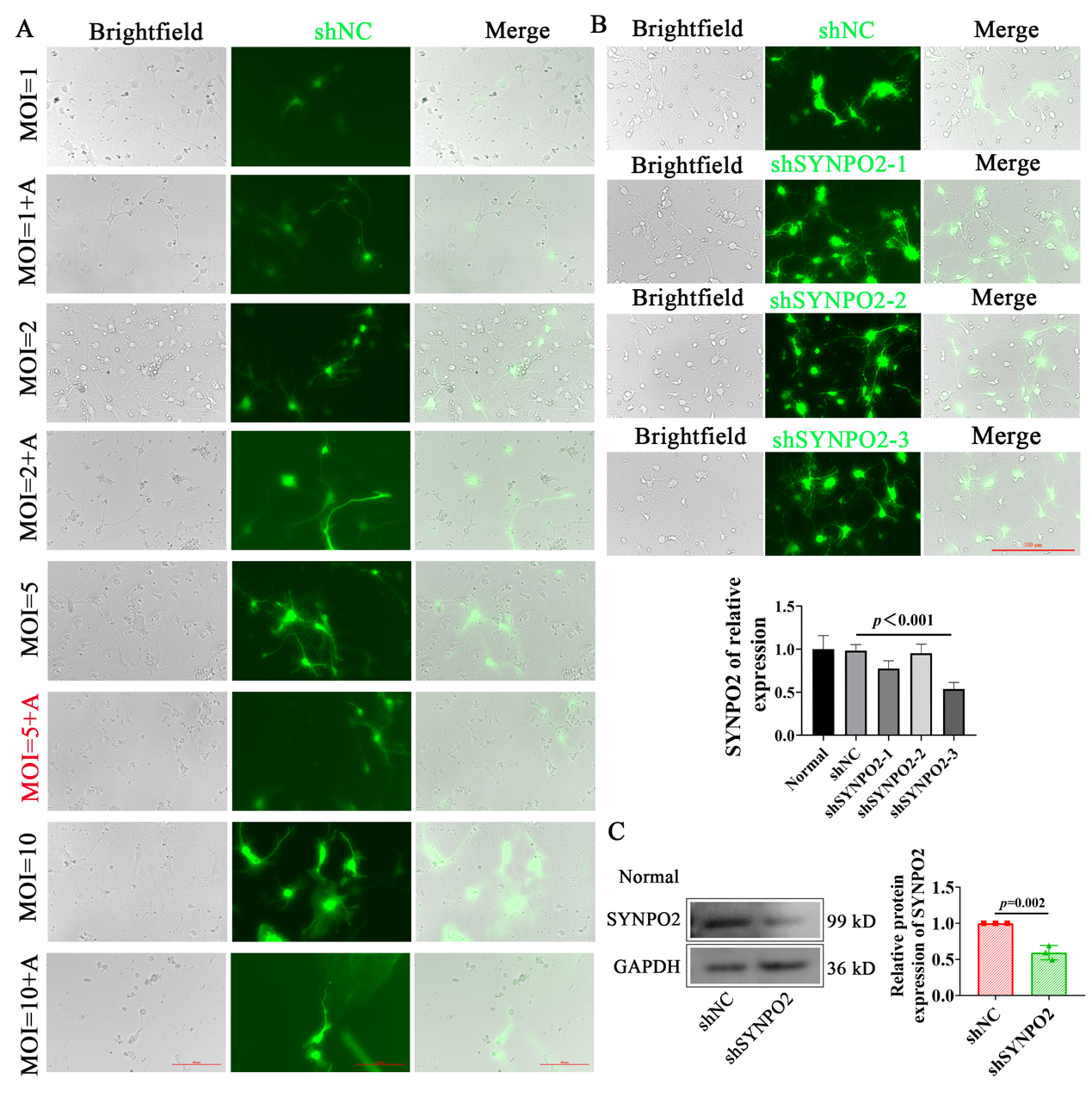
**

**Fig. S3 Transfection efficiency of lentivirus in cortical neurons.** **A.** Infection efficacy of shNC lentivirus under different conditions, scale bar=100 μm. **B.** Infection efficacy of different fragments of shSYNPO2, scale bar=100 μm. **C.** Expression levels of SYNPO2 in normal neurons infected with shNC and shSYNPO2. Bar graphs display mean ± SD of independent biological replicates. n = 3 wells, two-sided unpaired one-way ANOVA (**B**), two-sided unpaired independent sample *t*-test (**C**). All experiments were repeated three times independently, with similar results. MOI, multiplicity of infection; A, infection reagent A.


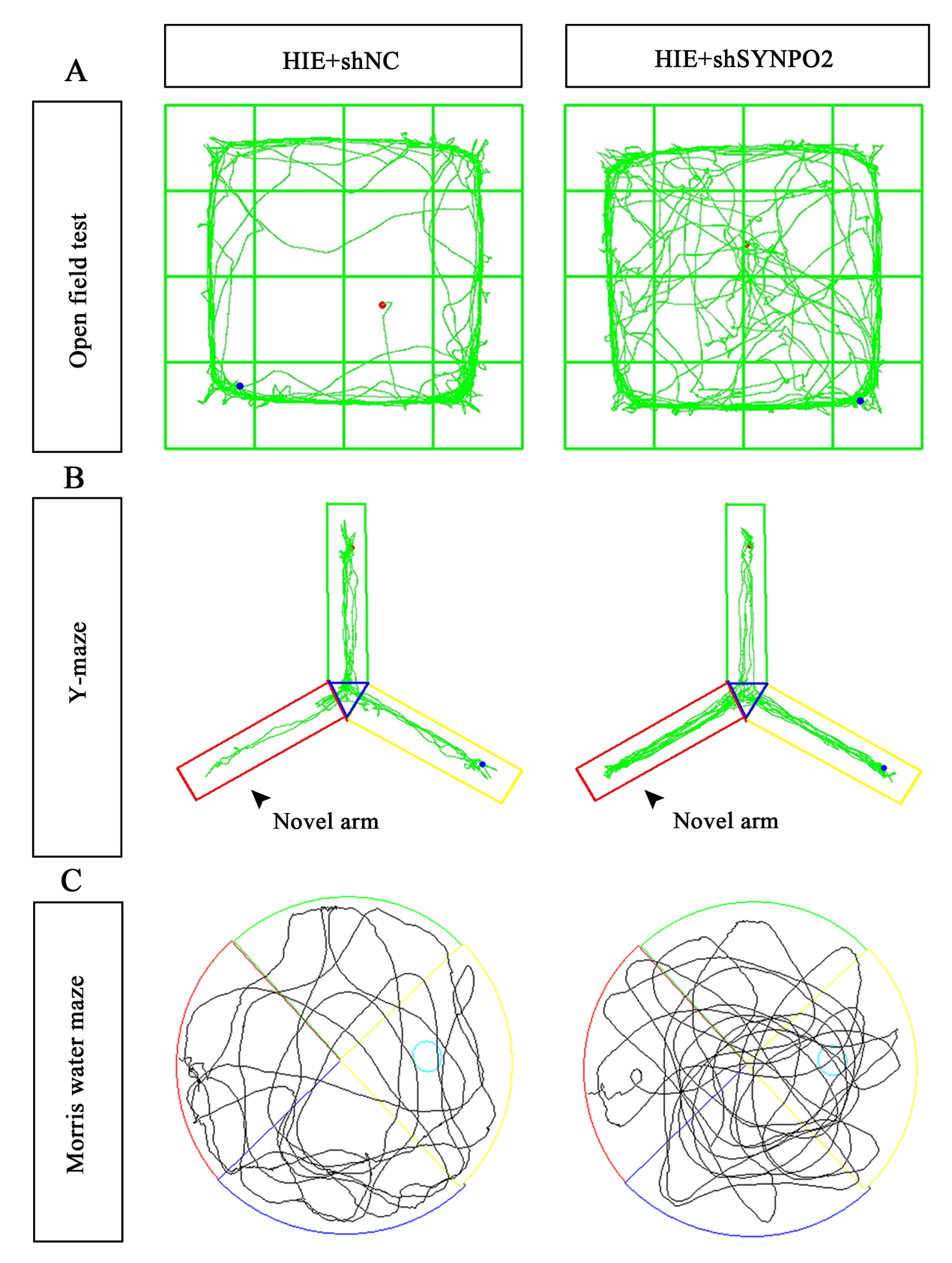


**Fig. S4 SYNPO2 interference alleviates anxiety and ameliorates neurological deficits in HIE rats.** **A-C.** Trajectory of the open field **(A)**, Y-maze **(B),** and Morris water maze **(C)** tests in HIE rats injected with lentivirus. n = 4-8 rats. HIE+shNC, rats injected with control interfering lentivirus before HIE modeling; HIE+shSYNPO2, rats injected with SYNPO2 interfering lentivirus before HIE modeling


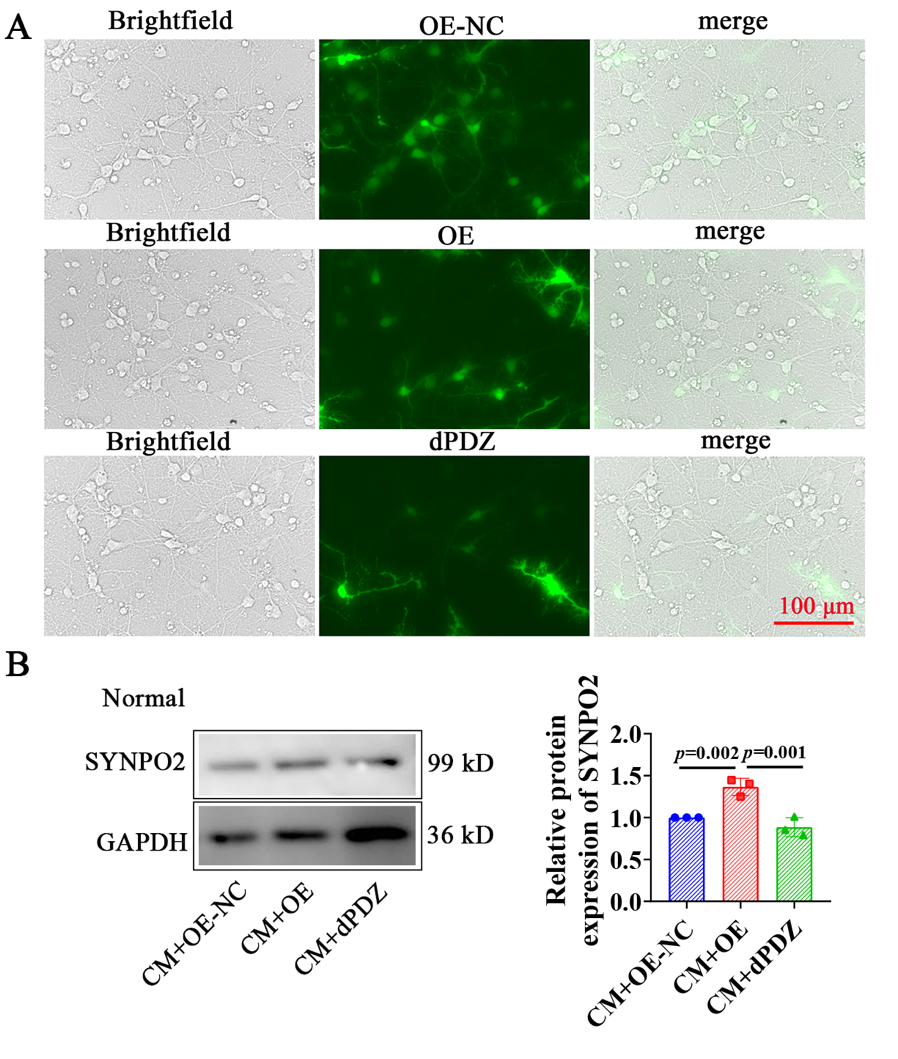


**Fig. S5 Lentiviral infection of neurons. A.** Infection efficacy of SYNPO2 lentivirus, scale bar=100 μm. **B.** Expression levels of SYNPO2 in normal neurons infected with SYNPO2+OE-NC, SYNPO2+OE and shSYNPO2+dPDZ. Bar graphs display mean ± SD of independent biological replicates. n = 3 wells, two-sided unpaired one-way ANOVA. All experiments were repeated three times independently, with similar results. CM+OE-NC, cortical neurons infected with SYNPO2 overexpressing control lentivirus before OGD and subjected to BMSC treatment; CM+OE, cortical neurons infected with SYNPO2 overexpressing lentivirus before OGD and with BMSC infusion; dPDZ, cortical neurons infected with the SYNPO2-dPDZ deletion mutant lentivirus before OGD and treated with BMSC-conditioned medium. MOI, multiplicity of infection; A, infection reagent A.


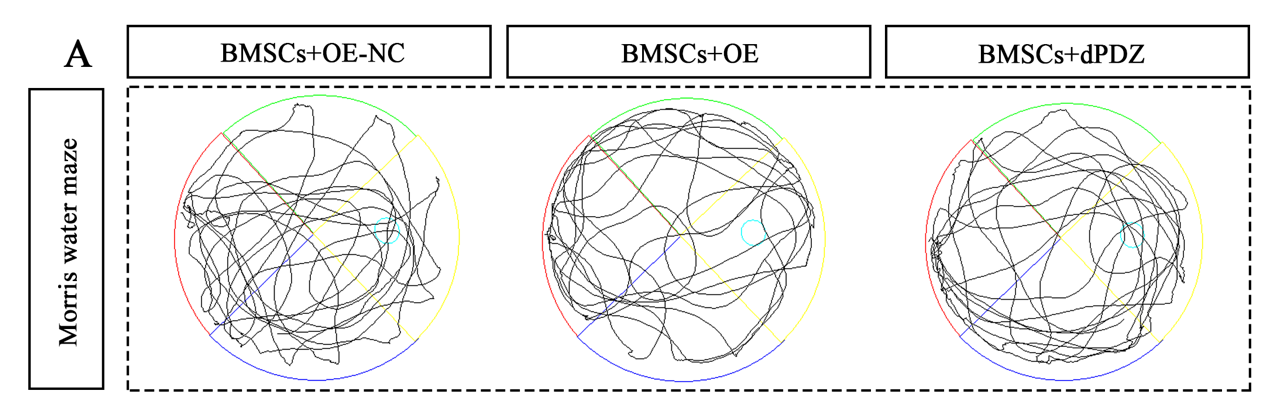


**Fig. S6 BMSCs improves neurological dysfunction in HIE rats by down-regulating SYNPO2. A.** Trajectory of water maze experiment of HIE rats in BMSCs+OE-NC, BMSCs+OE and BMSCs+dPDZ groups. n = 4-6 rats. BMSCs+OE-NC, rats injected with SYNPO2 overexpressing control lentivirus before HIE model establishment and BMSC treatment; BMSCs+OE, rats injected with SYNPO2 overexpressing lentivirus before HIE model establishment and BMSC treatment; BMSCs+dPDZ, rats injected with SYNPO2-dPDZ deletion mutant lentivirus before HIE model establishment and BMSC treatment.
