# Supplementary figures and images for: Bone marrow mesenchymal stem cells alleviate neurological dysfunction by reducing autophagy damage via downregulation of SYNPO2 in neonatal hypoxic–ischemic encephalopathy rats
Source: Cell Death Dis. 2025 Feb 25;16(1):131. doi: 10.1038/s41419-025-07439-w (PMC11862179; doi:10.1038/s41419-025-07439-w)

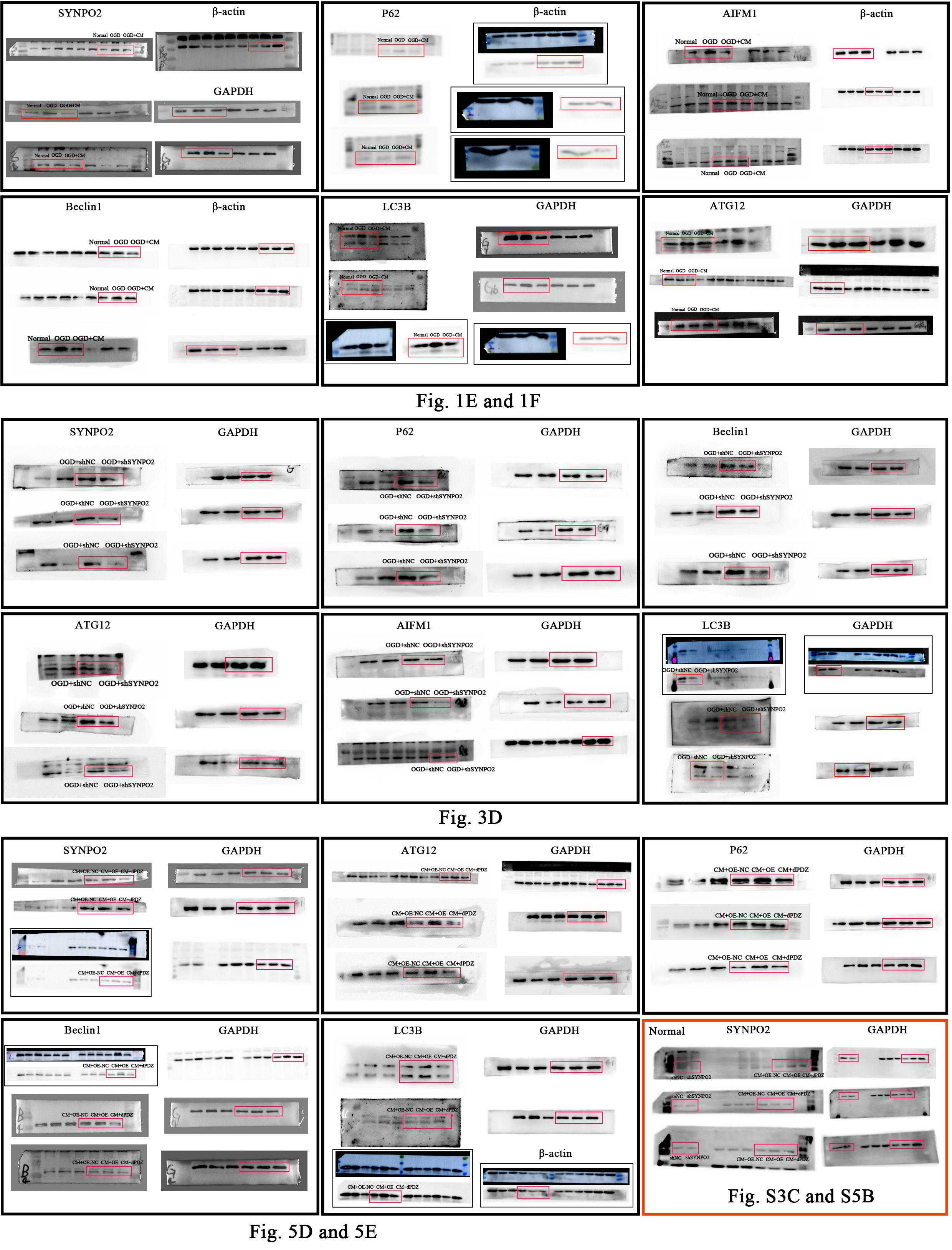

Supplement: Supplementary file 2 — Original data for WB [file 41419_2025_7439_MOESM2_ESM.tif]
